# Association of systemic immune-inflammation index with type 2 diabetes mellitus and its prognostic significance: a systematic review and meta-analysis

**DOI:** 10.3389/fendo.2025.1572089

**Published:** 2025-10-09

**Authors:** Ru Xu, Lele Jiang

**Affiliations:** Department of Clinical Laboratory, Beilun People’s Hospital, Ningbo, Zhejiang, China

**Keywords:** type 2 diabetes mellitus, systemic immune-inflammation index, association, prognosis, meta-analysis

## Abstract

**Background:**

The systemic immune-inflammation index (SII), a novel biomarker, may be associated with type 2 diabetes mellitus (T2DM). This study aimed to investigate the relationship between SII and T2DM, as well as its prognostic value.

**Methods:**

A comprehensive search of PubMed, Embase, Cochrane Library, Web of Science, CNKI, VIP, Wanfang, and Biomedical Literature Database was conducted to identify eligible studies published up to October 26, 2024. Relative risk (RR), odds ratio (OR), and hazard ratio (HR), along with their 95% confidence intervals (CIs), were extracted and synthesized. Statistical analyses were performed using Stata 15.1 software.

**Results:**

21 studies were included. SII was associated with an increased risk of major adverse cardiovascular events (MACE) and mortality. Each one-unit standard deviation (SD) increase in SII was positively correlated with MACE risk (OR/HR = 1.07; 95% CI: 1.04-1.10; *P* < 0.001). However, no significant association was found between SII and diabetic retinopathy. Regarding glucose metabolism abnormalities and diabetic nephropathy, high SII was significantly associated with increased risk (*P* < 0.05).

**Conclusions:**

Elevated SII is associated with an increased risk of MACE, mortality, diabetic nephropathy and glucose metabolism abnormalities but shows no significant correlation with diabetic retinopathy.

## 1 Introduction

In 2021, there were 529 million individuals living with diabetes globally, with type 2 diabetes mellitus (T2DM) accounting for approximately 96.0% of all cases (1). By 2050, the global number of diabetes cases is projected to exceed 1.31 billion, making its continued rise one of the major public health challenges (1). Researchers have suggested that diabetes is largely preventable, especially through early detection and management, which may enable primary prevention. In addition, diabetic patients often experience various complications, such as cardiovascular diseases, nephropathy, retinopathy, and neuropathy, which can lead to severe disability and premature death (2), placing a significant burden on families and society. Therefore, exploring effective biomarkers to assess diabetes risk, monitor disease progression, and predict prognosis is crucial.

A substantial body of research indicates that chronic tissue inflammation has been recognized as a hallmark feature of T2DM, reflecting the complex interplay between immune responses and metabolic dysfunction (3). This persistent inflammatory state not only promotes the progression of T2DM but also complicates its management by worsening insulin resistance and impairing beta-cell function (4). Given the critical role of inflammation in T2DM, there is a growing demand for biomarkers that can more accurately reflect patients’ systemic inflammatory status. The Systemic Immune-Inflammation Index (SII) is a novel composite biomarker that reflects the balance between host immune and inflammatory status. It was first proposed by Hu et al. in 2014 in the context of hepatocellular carcinoma prognosis (5). SII is calculated using the formula:SII = Platelet count × Neutrophil count/Lymphocyte count. The rationale for this index is grounded in the observation that elevated neutrophils and platelets are associated with enhanced pro-inflammatory responses and tumor progression, while decreased lymphocytes indicate impaired immune surveillance (6, 7). Thus, SII integrates both innate immunity (via neutrophils and platelets) and adaptive immunity (via lymphocytes), providing a more holistic marker of systemic inflammation than traditional indicators such as Neutrophil/lymphocyte ratio or platelet/lymphocyte ratio. Recent studies have highlighted the unique value of the SII in both tumor-related (8, 9) and non-tumor diseases (10, 11), where it is widely used to assess inflammation and explore its clinical implications. A cross-sectional study published by Guo et al. (12) demonstrated that a high SII level is independently associated with an increased risk of diabetic kidney disease. Luo et al. (13) reported that in patients with acute myocardial infarction and conexisting diabetes, a higher SII index was an independent predictor of mortality. Furthermore, a cohort study involving 2,018 individuals with diabetes or prediabetes found that elevated SII was associated with an increased risk of all-cause and cardiovascular mortality (14). However, the clinical application of SII is not without limitations. It may be influenced by acute infections, hematological disorders, and systemic conditions, leading to non-specific elevations (6, 7). Furthermore, the cutoff values of SII vary across studies and populations, reflecting the lack of standardized thresholds (6). Therefore, interpretation of SII should be context-dependent and validated within specific disease populations (15). Although some studies have highlighted the prognostic significance of SII, it is regrettable that there currently lacks a comprehensive meta-analysis to systematically evaluate the impact of SII on Type 2 Diabetes Mellitus. Therefore, this study aims to explore the association between SII and diabetes, as well as its prognostic significance, through a systematic review and meta-analysis of existing literature. The findings are expected to provide a scientific basis for early prevention, risk assessment, and personalized treatment strategies for diabetes.

## 2 Method

This study was conducted in accordance with the Preferred Reporting Items for Systematic Reviews and Meta-Analyses (PRISMA) guidelines (16) and the Meta-analysis of Observational Studies in Epidemiology (MOOSE) guidelines (17–19). The protocol was registered on the PROSPERO platform (Registration No. CRD42024607071).

### 2.1 Search strategy

A comprehensive search was conducted in four English databases (PubMed, Embase, Cochrane Library, Web of Science) and four Chinese-language databases (CNKI, VIP, Wanfang, and Biomedical Literature Database). The search covered all records from database inception to October 26, 2024, and was limited to studies published in English or Chinese. A combination of MeSH terms and free-text terms was used, with the following keywords: (Diabetes Mellitus OR diabetes OR diabetic OR diabetes OR diabet* OR T2D OR IDDM OR NIDDM) AND (systemic immune-inflammation index OR SII). The detailed search strategy is provided in **Supplementary Data Sheet 1.**

### 2.2 Literature selection

The included studies met the following criteria (1): Study Population: Adult patients diagnosed with T2DM (2); Exposure Factor: Studies reporting the SII levels and their association with glucose metabolism abnormalities or clinical prognosis in diabetic patients (3); Outcomes: Associations with glucose metabolism abnormalities or clinical outcomes, including major adverse cardiovascular events (MACE), all-cause mortality, cardiovascular mortality, myocardial infarction, cerebral infarction, severity of coronary artery stenosis, target vessel revascularization, renal mortality, cancer mortality, glucose metabolism abnormalities, diabetic retinopathy, diabetic nephropathy, and peripheral neuropathy (4); Data Reported: Studies reporting relative risk (RR), odds ratio (OR), hazard ratio (HR) with corresponding 95% confidence intervals (CIs), or providing raw data sufficient for their calculation.

The following studies were excluded (1): Reviews, case reports, study protocols, or conference abstracts; (2) Clinical trials, animal studies, or *in vitro* studies. Clinical trials were excluded because, to the best of our knowledge, no peer-reviewed clinical trial has yet been published that specifically investigates the association between SII and diabetic complications in patients with type 2 diabetes; (3) Duplicate or unavailable full-text studies; (4) The outcome indicators could not be extracted.

Two reviewers, XR and JLL, independently screened the literature based on the above criteria. Disagreements during the selection process were resolved through discussion or consultation with a third reviewer, LCY.

### 2.3 Data extraction and quality assessment

Two reviewers (XR and JLL) independently extracted data from the included studies, including: First Author, Year, Country, Study Design, Sample Size, Sex, Age, Disease Background, Duration of Diabetes, SII Cutoff, Follow-Up Time, Study Outcomes, Adjustments and Confounders. The quality of the included studies was independently assessed by the two reviewers (XR and JLL) using the Newcastle-Ottawa Scale (NOS) for evaluating non-randomized studies in meta-analyses (20). The NOS is applicable for both retrospective studies (selection, comparability, and exposure) and prospective studies (selection, comparability, and outcome). For retrospective studies, eight criteria were evaluated: adequacy of case definition, representativeness of cases, selection of controls, definition of controls, comparability of cases and controls based on study design or analysis, ascertainment of exposure, consistency in ascertainment between cases and controls, and comparability of non-response rates. For prospective studies, the assessment included: representativeness of the exposed cohort, selection of the non-exposed cohort, ascertainment of exposure, demonstration that the outcome of interest was not present at baseline, comparability of cohorts based on study design or analysis, assessment of outcomes, sufficiency of follow-up duration for outcome occurrence, and adequacy of cohort follow-up. A maximum score of 2 points can be awarded for comparability, while the other seven aspects can each receive a maximum of 1 point, resulting in a total score of 9. Studies with a total score of ≥6 are defined as high-quality studies. For cross-sectional studies, quality was assessed using the checklist from the Joanna Briggs Institute (JBI) Centre for Evidence-Based Health Care in Australia. The JBI checklist consists of 8 items, and evaluators are required to judge each item as “Yes,” “No,” or “Unclear.” Studies were considered high quality if ≥80% of the responses were rated as ‘Yes’ (21).

### 2.4 Data synthesis and statistical analysis

The primary outcomes of interest were major adverse cardiovascular events (MACE) and mortality-related events. Secondary outcomes included glucose metabolism abnormalities, diabetic retinopathy, diabetic nephropathy, and peripheral neuropathy.

Heterogeneity across studies was first assessed using the chi-squared (χ²)-based Q test and the I² statistic. Meta-analysis was then conducted using Stata 15.1. If no significant heterogeneity was observed (I² < 50% and *P* > 0.1), a fixed-effects model (Mantel-Haenszel method) was applied. Otherwise, a random-effects model (DerSimonian-Laird method) was used. Subgroup and meta-regression analyses were performed based on SII cutoff values, age, sex, sample size, and study type to explore the extent and sources of heterogeneity among studies. Sensitivity analysis was conducted to assess the robustness of the meta-analysis results. Publication bias was evaluated using funnel plots and Egger’s or Begg’s test (for analyses including ≥5 studies). If significant publication bias was detected, the trim-and-fill method was applied to measure the potential impact on the results.

## 3 Results

### 3.1 Literature screening results & flowchart

**Figure 1:**
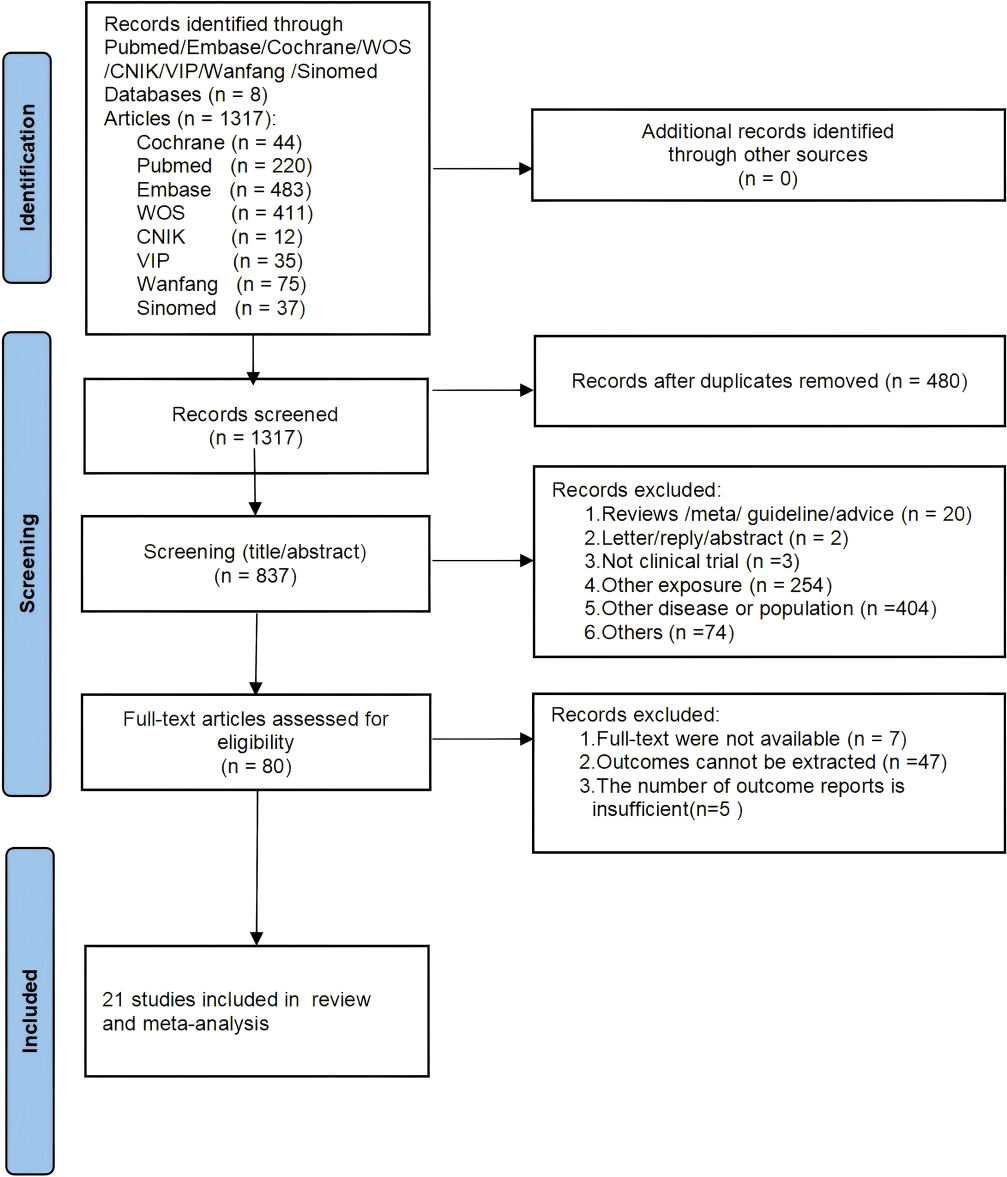
PRISMA flowchart of study selection process.

A total of 1,317 articles were identified through the initial database search, with no additional records found through reference scanning. After removing duplicates, 837 articles remained for titles and abstracts screening. Of these, 757 were excluded for not meeting the inclusion criteria, leaving 80 articles for full-text review. Finally, 21 studies were included in this meta-analysis (12, 13, 15, 22–39). The literature selection process is shown in **Figure 1**.

### 3.2 Basic characteristics of included studies

The 21 included studies were conducted in four countries: 18 from China (12, 13, 15, 22–25, 27, 29–31, 34–39), one from Switzerland (33), one from Romania (28), and one from Saudi Arabia (26). Among them, 3 were prospective studies, 11 were cross-sectional studies, and 7 were retrospective studies, involving a total of 192,679 patients. Of these, 121,233 were male and 71,446 were female, with a mean age ranging from 45 to 71 years. Detailed characteristics of the included studies are provided in **Supplementary Table 1**.

### 3.3 Quality assessment

Quality assessment of the retrospective studies using the NOS scale indicated that all 7 included studies scored ≥6 points (**Supplementary Table 2-1**). In the Comparability of cohorts based on study design analysis domain, 3 studies scored 2 points, while 4 studies scored 1 point due to not controlling for age, potentially introducing bias. In the Ascertainment of exposure domain, 6 studies scored 1 point, whereas 1 study scored 0 points because exposure was determined solely from medical records. Regarding the Nonresponse rate domain, 5 studies scored 1 point, whereas 2 studies scored 0 points due to the lack of description. Similarly, the prospective studies using the NOS scale also demonstrated good quality, with all 3 included studies scored ≥ 6 points (**Supplementary Table 2-2**). In the Comparability domain, 2 studies scored 2 points, while 1 study scored 1 point for not adjusting for age. For the domains ‘Was follow-up long enough for outcomes to occur’ and ‘Adequacy of follow-up of cohorts’, two studies scored 1 point, while one study scored 0 due to insufficient follow-up description. Quality assessment of the cross-sectional studies using the JBI checklist showed that among the 11 included studies, 8 were rated as “High” quality and 3 as “Moderate” quality (**Supplementary Tables 2**, **Supplementary Table 3**).

### 3.4 Meta-analysis results

#### 3.4.1 Prognostic value for MACE

**Figure 2:**
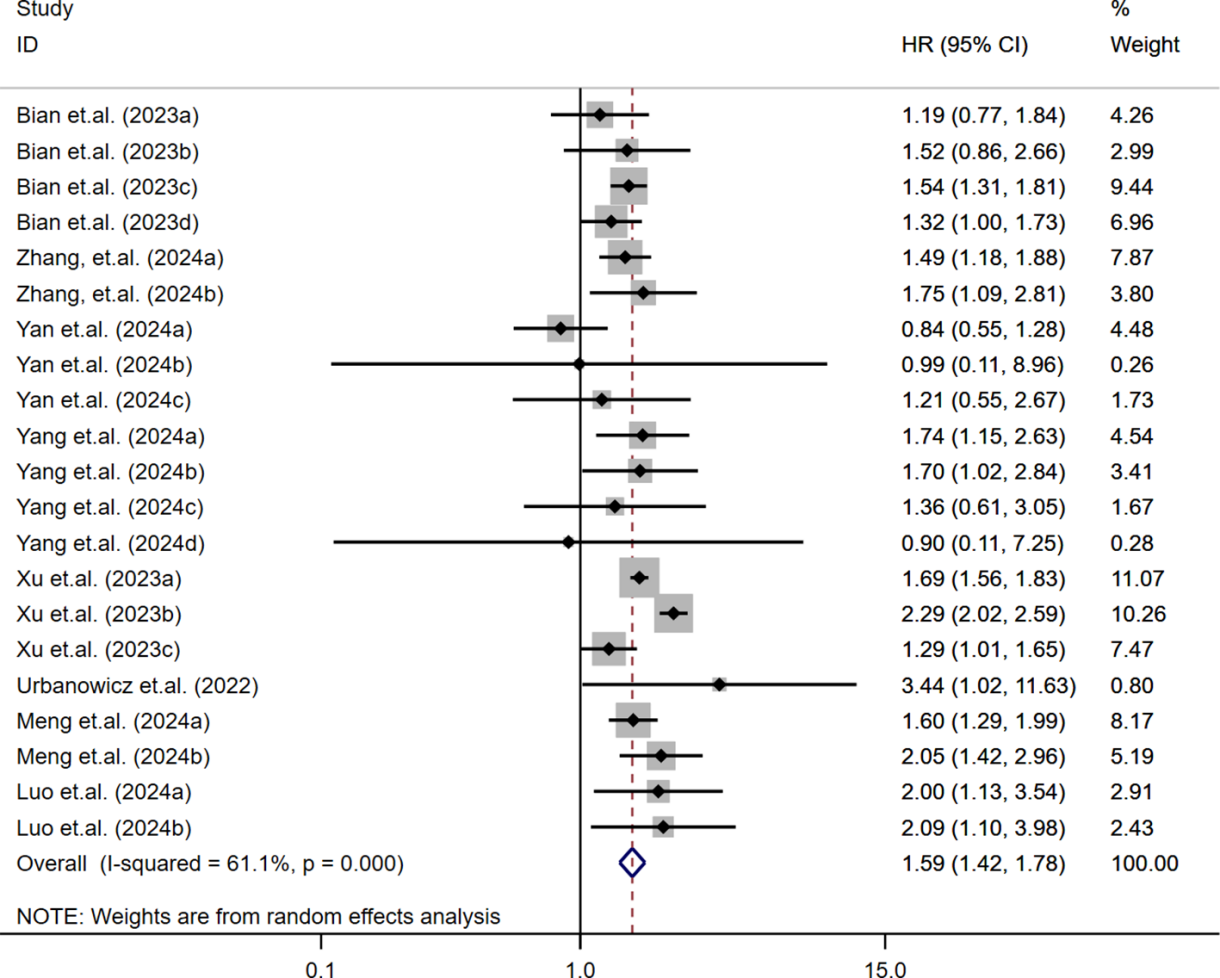
Association between high SII and risk of MACE.

The relationship between SII and MACE in diabetic patients was analyzed using a random-effects model due to moderate heterogeneity (I² = 61.1%, τ² = 0.0282, *P* < 0.001) (13, 15, 31, 33, 35–38). The results indicated that higher SII levels were significantly associated with an increased risk of MACE in patients with T2DM (HR = 1.59; 95% CI: 1.42-1.78; *P* < 0.001). As shown in **Figure 2**, this association was consistent across most included studies, with 18 out of 21 effect estimates favoring a positive correlation between elevated SII and MACE risk. Overall heterogeneity was moderate (I² = 61.1%), indicating some variation but not enough to obscure the general trend. These findings suggest that elevated SII may serve as a moderately strong predictor for MACE events in this population.

**Figure 3:**
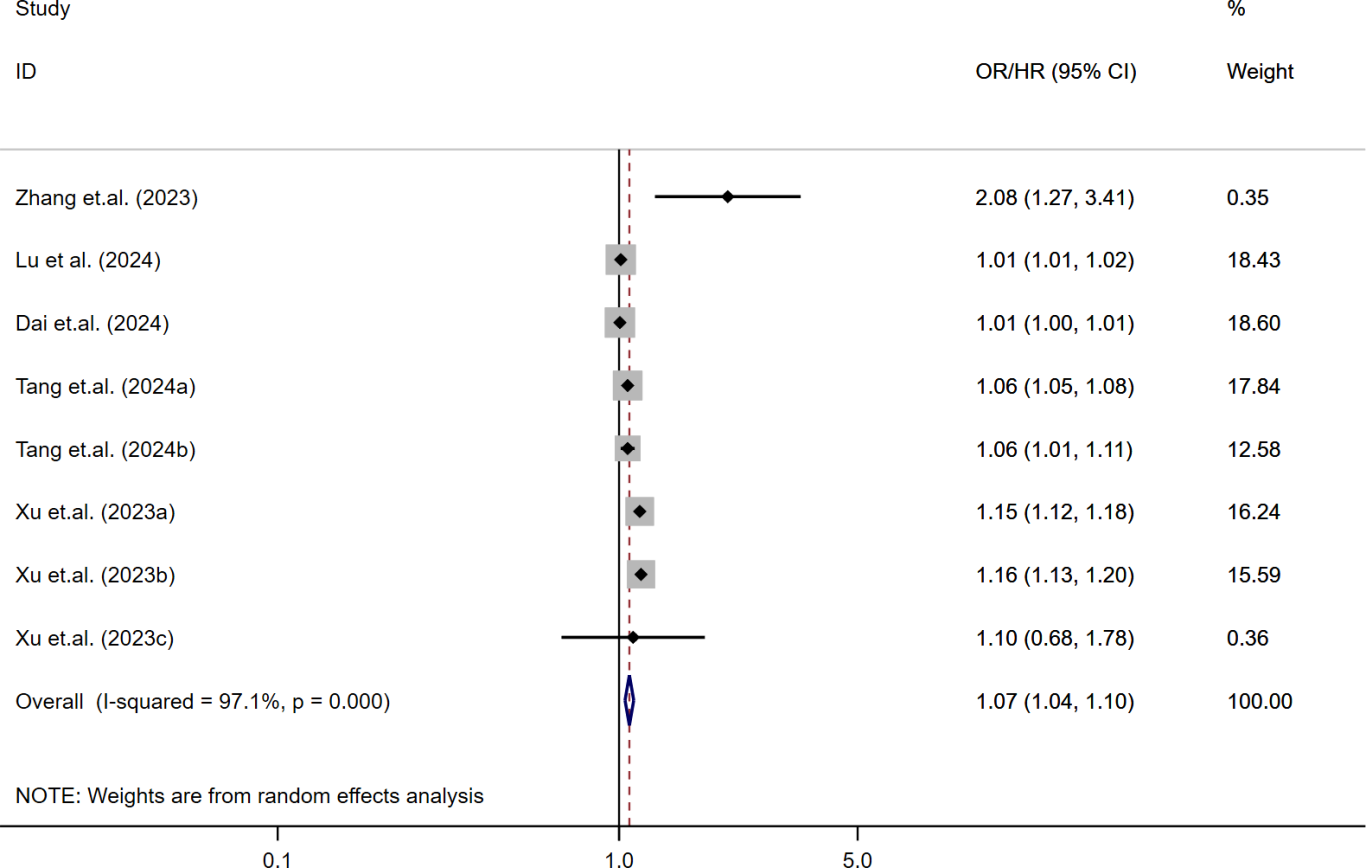
Per-SD increase in SII and risk of MACE.

The relationship between continuous SII levels and MACE risk in diabetic patients was assessed across nine studies (23–25, 32, 35). Due to substantial heterogeneity (I² = 97.1%, τ² = 0.0012, *P* < 0.001), a random-effects model was used. The pooled analysis indicated that each 1-SD increase in SII was significantly associated with increased MACE risk (OR/HR = 1.07; 95% CI: 1.04-1.10; *P* < 0.001). These findings, shown in **Figure 3**, suggest a consistent positive association across studies despite variability in effect sizes.

#### 3.4.2 Prognostic value for mortality rate

**Figure 4:**
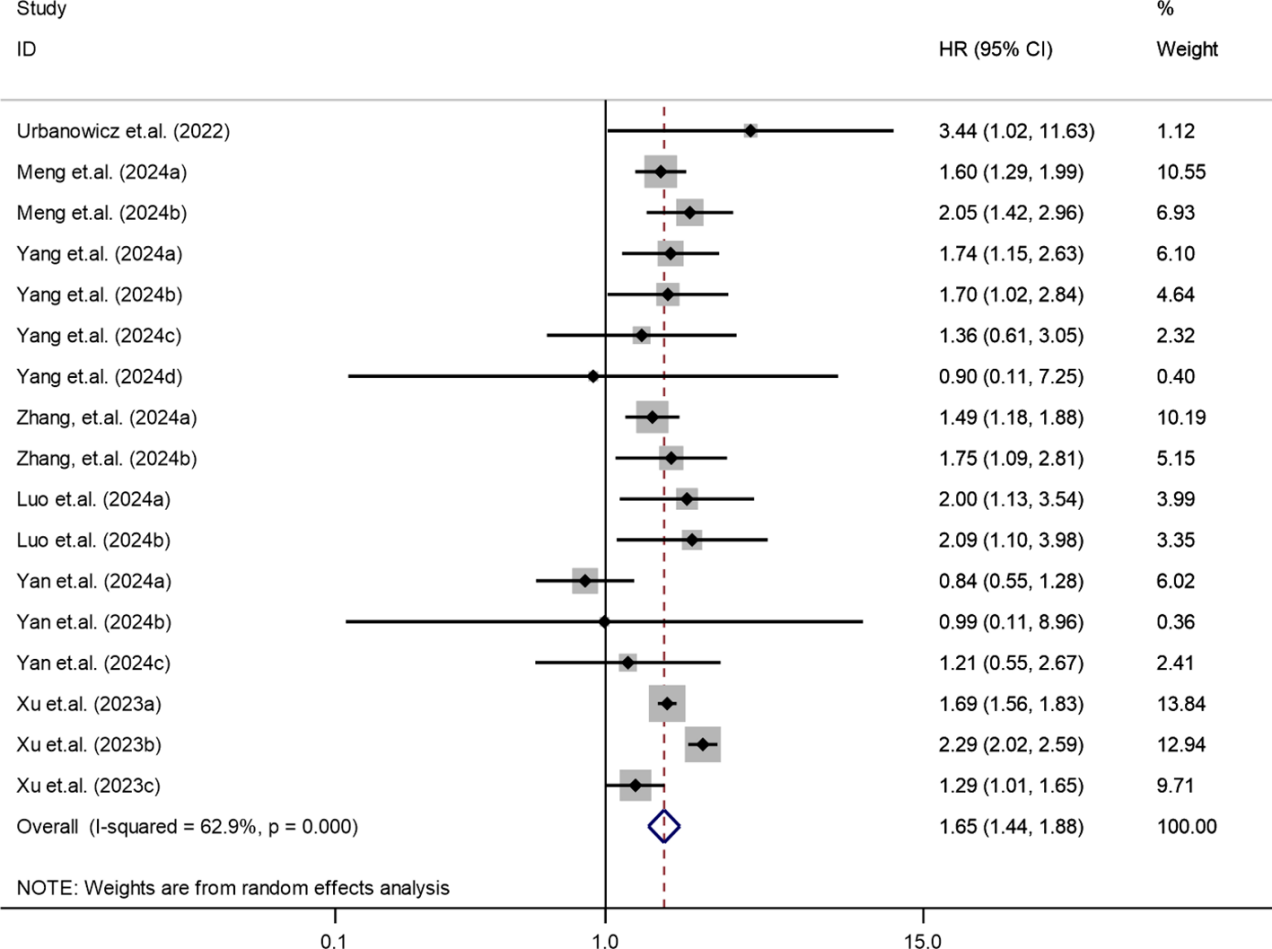
Association between elevated SII and mortality risk in T2DM patients.

The association between SII and mortality risk was evaluated across multiple studies (13, 31, 33, 35–38). Moderate heterogeneity was detected (I² = 62.9%, τ² = 0.0322, P < 0.001), warranting the use of a random-effects model. The pooled results showed that patients with higher SII levels had a significantly elevated risk of mortality (HR = 1.65; 95% CI: 1.44-1.88; *P* < 0.001). These findings, illustrated in **Figure 4**, highlight the prognostic significance of SII for adverse survival outcomes.

#### 3.4.3 Association of SII with abnormal glucose metabolism

**Figure 5:**
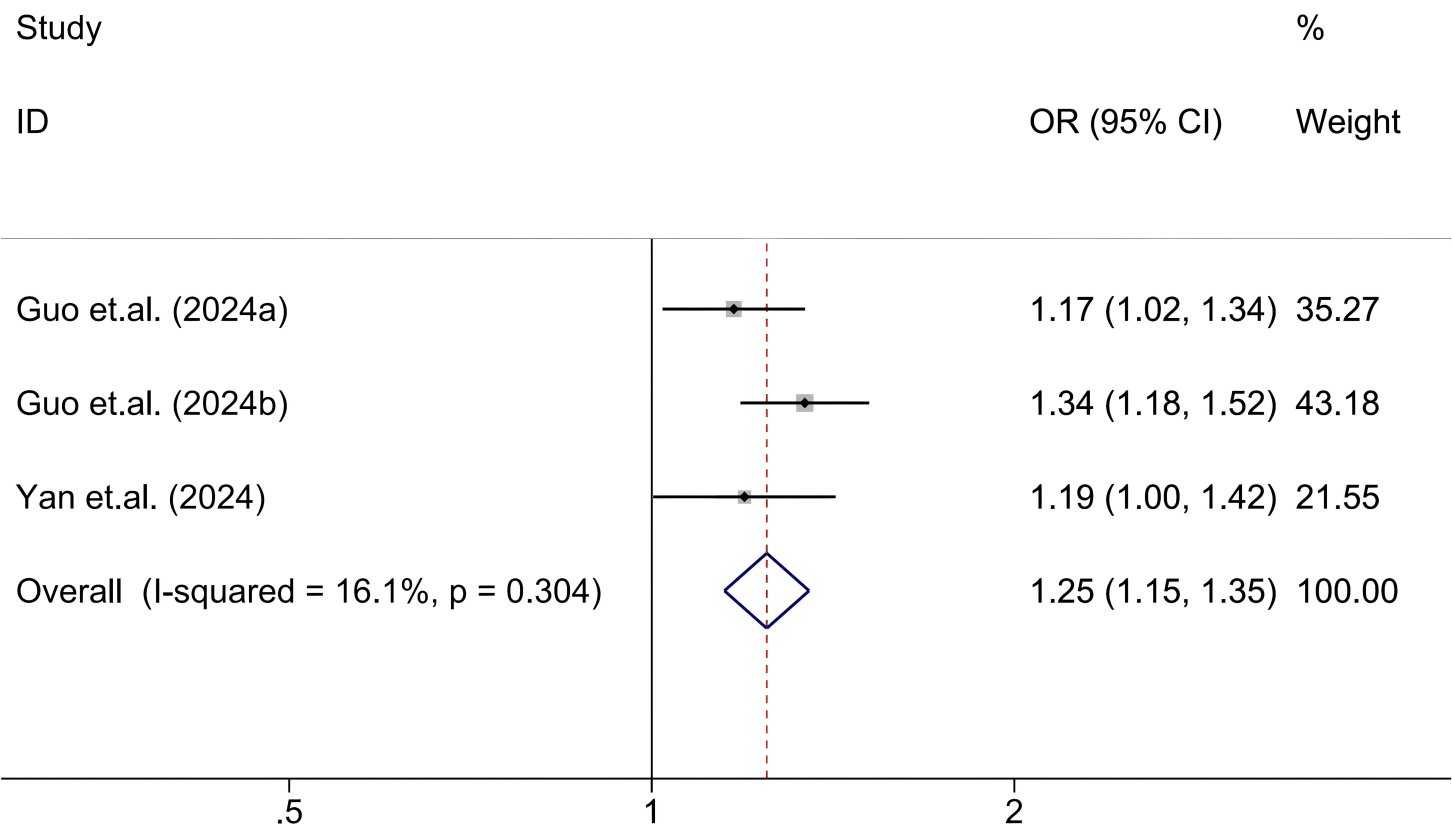
Association between high SII and abnormal glucose metabolism.

The association between SII and abnormal glucose metabolism was assessed based on three cohorts from two independent studies (29, 36). Given the low heterogeneity across studies (I² = 16.1%, *P* = 0.304), a fixed-effects model was used. The pooled results revealed that elevated SII levels were significantly associated with increased odds of abnormal glucose metabolism (OR = 1.25; 95% CI: 1.15-1.35; *P* < 0.001), indicating a consistent and directionally positive relationship across all included cohorts (**Figure 5**).

#### 3.4.4 Prognostic value for diabetic retinopathy

The relationship between SII and diabetic retinopathy was analyzed (28, 34). A fixed-effects model was used for the meta-analysis due to no observed heterogeneity (I² = 0.0%, *P* = 0.433).The results showed that a one standard deviation (SD) increase in SII was marginally non-significant in relation to diabetic retinopathy (OR = 1.001; 95% CI: 1.000-1.003; *P* = 0.026). The findings are illustrated in **Supplementary Figure 1**.

#### 3.4.5 Prognostic value for diabetic nephropathy

The relationship between SII and diabetic nephropathy was analyzed based on two studies (12, 39). A fixed-effects model was used due to low heterogeneity (I² = 16.8%, *P* = 0.273). The results indicated significant association between SII and diabetic nephropathy (OR = 1.55; 95% CI: 1.26–1.90; *P* < 0.001). The findings are displayed in **Supplementary Figure 2**.

### 3.5 Subgroup and regression analyses

Subgroup and meta-regression analyses were performed based on Age, Sex, Study Design, Sample Size, and SII cutoff values to explore the potential sources of heterogeneity for the primary outcomes (MACE and mortality). The results are summarized in **Supplementary Table 3**.

In the subgroup analyses, elevated SII were significantly associated with an increased risk of MACE and mortality across subgroups defined by Sex (Male or Female), Study Design (Cross-sectional, Prospective, or Retrospective studies), Sample Size (>5000 or <5000), and various Cut-off levels of SII (<600, >700, >900). For the Age subgroup, a significant association between SII and an increased MACE risk was observed when the average age was >50 years (HR = 1.65; 95% CI: 1.48–1.83; *P* < 0.01), while no significant association was observed in individuals aged <50 years (HR = 0.91; 95% CI: 0.63–1.31; *P* = 0.621). Similarly, for mortality, high SII was significantly associated with increased risk in the >50 age group (HR = 1.74; 95% CI: 1.54–1.97; *P* < 0.01) but not in the <50 age group (HR = 0.91; 95% CI: 0.63–1.31; *P* = 0.621). These findings suggest that Age is a key factor influencing the primary outcomes and may contribute to heterogeneity due to differing associations across age groups. Regarding the SII cutoff levels, no significant association with MACE was observed when the cutoff was between 600 and 700 (HR = 1.29; 95% CI: 0.96–1.74; *P* = 0.087). However, significant associations were observed when the cut-off was <600 (HR = 1.45; 95% CI: 1.28–1.65; *P* < 0.01), >700 (HR = 1.71; 95% CI: 1.40–2.09; *P* < 0.01), or >900 (HR = 1.78; 95% CI: 1.50–2.11; *P* < 0.01). A similar patterns was observed for mortality outcomes, SII cut-offs >700 or >900 were significantly associated with increased risk, but no significant association was found for cut-offs between 600 and 700. These variations in the associations across different SII cut-off ranges suggest that the Cut-off of SII is likely an important source of heterogeneity for both MACE and mortality outcomes. The regression analysis revealed that age, sex, study design, sample size, and the SII cutoff value contributed to heterogeneity in the main outcomes (MACE and mortality) (regression *P* < 0.05) **Supplementary Table 3**.

### 3.6 Sensitivity analysis

Sensitivity analyses were performed for the primary outcomes, MACE and mortality, by sequentially excluding each study to assess its impact on the pooled results. The analysis demonstrated that no single study significantly influenced the overall pooled results, indicating that the findings of this meta-analysis are relatively robust. The results of the sensitivity analyses are shown in **Supplementary Figures 3**, **Supplementary Figures 4** .

### 3.7 Publication bias

To ensure the validity of the meta-analysis results, funnel plots, Egger’s test, and Begg’s test were used to assess publication bias for the primary outcomes. No significant publication bias was detected for MACE when SII was analyzed as a categorical variable (P = 0.220). However, significant publication bias was onserved when SII was treated as a continuous variable (P < 0.01). Using the trim-and-fill method, five additional studies were imputed, and the conclusions remained unchanged, further confirming the robustness of our findings. For mortality, no significant publication bias was observed (P = 0.393). The funnel plots are shown in **Supplementary Figures 5**-**Supplementary Figures 6**.

## 4 Discussion

This meta-analysis included 21 studies, all involving patients with T2DM. Consistent with previous research, our findings provide additional evidence supporting the prognostic value of SII in T2DM patients. Specifically, higher SII levels were positively associated with an increased risk of adverse clinical outcomes, including MACE, all-cause mortality, cardiovascular mortality, target vessel revascularization, cancer-related mortality, renal mortality, diabetic nephropathy, and glucose metabolism abnormalities. However, no significant association was found between SII and diabetic retinopathy.

High SII levels are associated with poor prognostic outcomes, such as MACE and cardiovascular mortality. Biologically, the inflammatory state reflected by elevated SII can promote atherosclerosis formation (40). Inflammatory cytokines stimulate vascular endothelial cells to express adhesion molecules such as intercellular adhesion molecule-1 (ICAM-1) and vascular cell adhesion molecule-1 (VCAM-1) (41, 42). These molecules facilitate the adhesion of monocytes to the vascular endothelium, allowing them to migrate beneath the endothelium, differentiate into macrophages, and engulf oxidized low-density lipoprotein (ox-LDL), forming foam cells (43), which are early components of atherosclerotic plaques. Simultaneously, platelet activation contributes to thrombogenesis, increasing the risk of cardiovascular events (44). For outcomes such as all-cause mortality and renal mortality, high SII levels may exert effects through direct or indirect organ damage (45, 46). For instance, in the kidneys, inflammatory responses can lead to the infiltration of inflammatory cells into the glomeruli, damaging the glomerular filtration barrier (47). Elevated SII levels are associated with adverse outcomes and may have potential value in risk stratification. For patients with elevated SII levels, more aggressive interventions, such as intensive lipid-lowering therapies and antiplatelet treatments, may be implemented to reduce the risk of adverse outcomes.

The relationship between SII and glucose metabolism abnormalities can be understood from the perspective of inflammation and immune dysregulation: From the inflammation response perspective, SII is an integrated marker reflecting the body’s inflammatory and immune status. In the development and progression of glucose metabolism abnormalities, inflammation plays a critical role (48). During chronic inflammation, inflammatory cytokines such as interleukin-6 (IL-6) and tumor necrosis factor-alpha (TNF-α) are elevated. These cytokines can interfere with insulin signaling pathways, leading to insulin resistance (49). For example, IL-6 can activate the JAK/STAT signaling pathway (50), reducing tyrosine phosphorylation of insulin receptor substrate (IRS) proteins, thereby impairing insulin signal transduction and reducing cellular insulin sensitivity. Components of SII, such as white blood cell counts, are closely related to inflammatory responses. Elevated SII levels may indicate a heightened inflammatory state, which could promote the occurrence of glucose metabolism abnormalities. From the immune cell dysfunction perspective, SII reflects the involvement of neutrophils, lymphocytes, and platelets. Under normal circumstances, immune cells contribute to maintaining glucose homeostasis. For instance, T cells within lymphocytes help regulate pancreatic β-cell function (51). However, elevated SII levels may indicate immune cell dysfunction. Neutrophils overactivation can lead to the release of reactive oxygen species (ROS), which can damage pancreatic β-cells (52). Additionally, abnormal platelet activation is often associated with vascular endothelial dysfunction, indirectly affecting glucose uptake and metabolism. Vascular endothelial cells play a crucial role in insulin-mediated glucose transport (52, 53). For clinicians, SII can serve as a potential risk assessment marker for glucose metabolism abnormalities. In T2DM patients with elevated SII levels, greater attention should be paid to changes in their glucose metabolism, and treatment plans may need to be adjusted, such as intensifying glycemic control. Moreover, a deeper understanding of the relationship between SII and glucose metabolism abnormalities could help identify new therapeutic targets. For example, developing drugs that modulate inflammatory responses or immune cell function may provide new avenues for managing glucose metabolism abnormalities.

Inflammatory responses can influence microvascular complications of diabetes, such as diabetic retinopathy (DR) and diabetic nephropathy (DN), through various pathways (53, 54). This study demonstrated that high SII levels were significantly associated with an increased risk of DN (OR = 1.55, 95% CI: 1.26-1.90, *P* < 0.001), a finding that both corroborates and extends earlier work (12, 55). Mechanistically, SII integrates neutrophil, platelet, and lymphocyte counts to reflect the balance between systemic inflammation and immune regulation. An elevated SII indicates neutrophilia and platelet activation alongside relative lymphopenia, denoting heightened inflammatory and pro−thrombotic activity. Neutrophils may injure glomerular endothelium and amplify inflammation via the release of reactive oxygen species and proteolytic enzymes; activated platelets interact with leukocytes through P−selectin/PSGL−1 and CD40L pathways, promoting their infiltration into the renal interstitium and local release of pro−inflammatory mediators, thereby exacerbating glomerular damage. Concurrent lymphocyte depletion implies weakened adaptive immune control, further perpetuating chronic inflammation (56). However, our study found no significant association between SII levels and DR in T2DM patients. This may be due to the body’s complex compensatory mechanisms. For instance, the body may upregulate anti-inflammatory factors or enhance cellular repair mechanisms to counteract inflammation-induced damage. Endothelial cells, when exposed to prolonged hyperglycemia, are activated, triggering inflammatory responses. Simultaneously, they may initiate protective programs, such as increasing nitric oxide (NO) production to maintain vascular dilation and mitigate inflammatory damage to microvasculature, potentially reducing the observable association between SII and microvascular complications. Genetic factors also play a critical role in the development of T2DM and its complications. Specific genes can influence an individual’s sensitivity to inflammation and susceptibility to microvascular complications. For example, polymorphisms in the angiotensin-converting enzyme (ACE) gene may alter the activity of the renin-angiotensin-aldosterone system (RAAS). Such genetic backgrounds may lead to enhanced regulation of the RAAS system, reducing the likelihood of microvascular complications like DR, even in individuals with elevated SII levels. Additionally, metabolic factors such as blood glucose, blood pressure, and blood lipids often interact in T2DM patients. Poor long-term glycemic control is a major risk factor for microvascular complications, but abnormalities in blood pressure and lipids also synergistically promote their progression. SII may reflect inflammation alone, while the intricate balance among these metabolic factors could obscure the relationship between SII and microvascular complications. For instance, when glycemic, blood pressure, and lipid levels are well-controlled within a certain range, high SII levels may not necessarily lead to significant microvascular damage. For clinicians, SII alone should not be relied upon when assessing the risk of DR in T2DM patients. A comprehensive evaluation should include multiple metabolic parameters, such as blood glucose, blood pressure, and blood lipids, alongside family history and other factors, to provide a holistic risk assessment for microvascular complications.

Notably, the absence of a significant association between SII and DR in our analysis may be explained by the lack of DR stage-specific stratification in the included studies. SII reflects both pro-inflammatory activity and immune suppression, representing a state of systemic immune imbalance. This is more relevant in PDR, which is characterized by widespread ischemia and systemic inflammatory activation, whereas NPDR involves primarily localized microvascular damage with limited systemic involvement. Therefore, SII may show a stronger association with PDR than NPDR. Additionally, variations in population characteristics, inadequate adjustment for confounders, and inconsistent SII cutoff values across studies may further contribute to these discrepancies. Future prospective studies with stage-specific DR classification and standardized analytical approaches are needed to clarify the clinical utility of SII in DR risk prediction and management.

Furthermore, since SII is a derived index based on routine blood cell counts (neutrophils, lymphocytes, and platelets), it can be conveniently incorporated into routine diabetes management without the need for additional laboratory testing. For T2DM patients with markedly elevated SII values (SII > 900), clinicians may consider enhanced cardiovascular risk monitoring-such as coronary CT angiography or cardiac biomarker screening-and initiate more aggressive lipid-lowering or anti-inflammatory interventions (statin therapy). Nonetheless, it is important to emphasize that SII should be used in conjunction with other established clinical indicators (eHbA1c, urine albumin-to-creatinine ratio) rather than as a standalone decision-making tool.

Additionally, subgroup analysis indicated that age and the SII cutoff value are likely key sources of heterogeneity. Among individuals aged >50 years, SII was significantly associated with an increased risk of MACE (HR = 1.65; 95% CI: 1.48–1.83; *P* < 0.01) and mortality (HR = 1.74; 95% CI: 1.54–1.97; *P* < 0.01). In contrast, no significant association was observed in those aged <50 years for either MACE (HR = 0.91; 95% CI: 0.63–1.31; *P* = 0.621) or mortality (HR = 0.91; 95% CI: 0.63–1.31; *P* = 0.621). This discrepancy may be attributed to age-related physiological and pathological changes. For example, older individuals are more likely to experience a decline in organ function, increased susceptibility to cardiovascular diseases, and altered immune system activity (57), which could make SII-related parameters more impactful on MACE and mortality outcomes. Conversely, younger individuals with relatively better physiological function may tolerate changes in SII-related parameters more effectively, resulting in a lack of significant association. This underscores the importance of considering age as a critical factor in both future research and clinical practice, and tailoring study designs or treatment strategies accordingly for different age groups. The variability in associations with primary outcomes across different Cut-off values of SII also highlights its potential as a heterogeneity source. Different cut-off values represent distinct thresholds of SII, potentially reflecting varying pathophysiological states. For instance, at higher cut-offs (>600–700), compensatory mechanisms or other unidentified factors may mitigate the associated risks (58), leading to no significant increase in risk.

Subgroup analyses revealed that age and SII cutoff values were likely the primary contributors to heterogeneity, with important biological implications. Notably, the association between elevated SII and increased risk of MACE and mortality was significant only among individuals aged >50 years. This age-related difference may be rooted in immunosenescence, a process characterized by overactivation of the innate immune system and impaired adaptive immunity in the elderly. Such dysregulation leads to chronic low-grade inflammation, which, when combined with endothelial dysfunction and declining organ reserve, may render older adults more vulnerable to the harmful effects of systemic inflammation. In contrast, younger individuals may exhibit stronger immune resilience and compensatory mechanisms, allowing them to better tolerate inflammatory burdens, thus attenuating the observed associations. Similarly, the variability in SII cut-off values across studies may reflect different degrees of systemic inflammatory activation. Higher thresholds (e.g., >700 or >900) likely capture more severe immune imbalance, directly promoting processes such as atherothrombosis and organ injury. Conversely, mid-range values (e.g., 600-700) may correspond to milder inflammation, which could be counteracted by protective physiological responses, leading to non-significant associations. These findings underscore the need for future studies to establish standardized, clinically relevant SII thresholds, and to consider age-related immune dynamics when applying SII in risk stratification and individualized treatment planning.

Conversely, in other cutoff ranges, the body may be more sensitive to SII-related changes, leading to a higher risk of primary outcomes. This suggests that when establishing and interpreting SII thresholds, careful consideration is required to evaluate their influence on key clinical endpoints, allowing for more accurate risk assessment and prediction. Despite the strength of our findings, potential publication bias remains a concern-particularly for the association between continuous SII levels and MACE. In our meta-analysis, Egger’s test and funnel plot asymmetry suggested the presence of small-study effects and publication bias. Although we applied the trim-and-fill method and found that the association remained statistically significant after adjusting for potentially missing studies, this statistical correction may not fully eliminate bias or account for unpublished negative results. Importantly, the presence of such bias could lead to an overestimation of the true effect size, thus weakening the confidence in the robustness of our conclusions. While trim-and-fill offers a useful sensitivity tool, it does not replace the need for rigorous reporting and balanced publication of both positive and null findings in the literature. Future studies with pre-registered protocols, larger sample sizes, and more consistent methodology are warranted to further validate our findings and reduce the impact of selective reporting. In contrast, across different subgroup classifications based on sex, study design, and sample size, elevated SII levels consistently showed significant associations with increased risks of MACE and mortality. This indicates that the relationship between SII and primary outcomes is relatively stable across these factors.

Although we conducted study quality assessments using the NOS and the JBI checklist, we did not perform meta-regression or sensitivity analyses based on study quality scores. This was primarily due to the limited number of studies for some outcomes, which restricted the statistical power for such subgroup comparisons. Nonetheless, the majority of included studies were of moderate to high quality, suggesting that the overall risk of bias due to low-quality studies may be limited. Future meta-analyses with larger sample sizes may benefit from incorporating study quality as a moderator in meta-regression models to better assess its impact on pooled estimates.

It should be noted that the optimal cut-off value of SII remains inconsistent across studies, which may hinder its direct clinical applicability. The variation in thresholds, study designs, and populations limits the ability to recommend a unified clinical use or integrate SII into established risk stratification systems. Therefore, while elevated SII levels appear to be associated with adverse outcomes in T2DM, the development of a standardized clinical algorithm remains premature. Nevertheless, SII provides valuable information on systemic inflammation and thrombosis pathophysiological domains not captured by traditional markers such as HbA1c or renal indices and may serve as a complementary biomarker to support comprehensive cardiovascular and renal risk assessment. Based on our subgroup analysis, an SII value exceeding 900 may serve as a potential “red alert” threshold, associated with significantly elevated risks of MACE and all-cause mortality. In clinical practice, this level of SII could prompt early intervention strategies, including (1) accelerated diagnostic screening such as cardiac stress testing, coronary artery calcium scoring, or renal function monitoring (2); intensified management of modifiable risk factors through optimized control of blood glucose, blood pressure, and LDL cholesterol; and (3) timely referral to cardiologists or endocrinologists for specialized care. Given its simplicity, accessibility, and cost-effectiveness, SII holds promise as an adjunctive tool in the personalized management of T2DM, particularly when integrated alongside established indicators in future standardized clinical pathways.

In addition, as artificial intelligence (AI) and machine learning (ML) based models are increasingly being used to enhance individualized risk prediction in chronic diseases, SII may serve as a valuable input variable for such algorithms. Its ability to reflect systemic inflammation and immune imbalance, both key drivers of cardiovascular and metabolic complications makes it a biologically meaningful and readily accessible biomarker. Future AI-driven risk stratification tools that incorporate SII alongside conventional metrics (e.g., HbA1c, age, renal function, and imaging data) may help improve early identification of high-risk T2DM patients, enabling more targeted preventive interventions.

## 5 Limitation

This meta-analysis has several limitations that should be acknowledged. First, the number of studies included was relatively small, particularly for analyses examining specific outcomes such as glucose metabolism abnormalities, diabetic nephropathy, and diabetic retinopathy. As such, the statistical power for these subgroups may be limited, and future updates incorporating additional studies will be necessary to validate these findings. Second, 18 of the 21 included studies were conducted in China, which may introduce geographic bias and limit the generalizability of our findings to broader ethnic or regional populations. Third, substantial heterogeneity was observed in the analysis of SII as a continuous variable, particularly for MACE outcomes. This heterogeneity likely stems from variations in study design, population characteristics, and SII cutoff values. Although we performed sensitivity and subgroup analyses to address this issue, some residual heterogeneity may remain.

Furthermore, all included studies were observational in nature, which precludes any definitive causal inference between elevated SII levels and adverse clinical outcomes. While SII was found to be significantly associated with increased risk of MACE, mortality, and diabetic nephropathy, its standalone predictive value is limited. We therefore recommend that SII be integrated with other established indicators-such as HbA1c, urine albumin-to-creatinine ratio, or imaging markers like coronary artery calcium scores-to enhance its prognostic utility in clinical practice. Additionally, we observed a discrepancy between our findings and those of Harley et al., who reported that SII was significantly elevated in patients with proliferative diabetic retinopathy (PDR). Our analysis did not find a significant association between SII and diabetic retinopathy overall, which may be due to (1) lack of DR stage stratification (NPDR vs. PDR) in our study, and (2) potential confounding from coexisting complications among the included populations. Future studies with prospective designs, stratified outcome definitions, and broader population diversity are warranted to confirm and refine these associations.

Given the broad range of outcomes included in this study (MACE, mortality, nephropathy, glucose metabolism abnormalities), this meta-analysis should be interpreted as an exploratory effort to map the prognostic relevance of SII across different T2DM complications, rather than a hypothesis-driven investigation focused on a single endpoint.

## 6 Conclusion

To the best of our knowledge, this is the first meta-analysis to examine the association between SII and clinical outcomes in patients with T2DM. Our findings indicate that elevated SII levels in T2DM patients are significantly associated with increased risks of MACE and mortality, but not with diabetic retinopathy. Additionally, high SII levels may be linked to an increased risk of glucose metabolism abnormalities and diabetic nephropathy. Based on these results, we recommend that future studies-particularly well-designed prospective investigations-be conducted to validate these findings and explore their potential applications in clinical practice.

## Data Availability

The original contributions presented in the study are included in the article/**Supplementary Material**. Further inquiries can be directed to the corresponding author.
