# Supplementary material for: Association of systemic immune-inflammation index with type 2 diabetes mellitus and its prognostic significance: a systematic review and meta-analysis
Source: Front Endocrinol (Lausanne). 2025 Oct 9;16:1572089. doi: 10.3389/fendo.2025.1572089 (PMC12548759; doi:10.3389/fendo.2025.1572089)
Supplement: Supplementary file 1 [file DataSheet1.docx]

# Supplementary Text 1 Literature search strategy

**1.Pubmed**

| Search number | Query |
| --- | --- |
| #1 | "Diabetes Mellitus"[Mesh] |
| #2 | ((((((((Diabetes Mellitus[Title/Abstract]) OR (diabetes[Title/Abstract])) OR (diabetic[Title/Abstract])) OR (diabetic[Title/Abstract])) OR (diabet*[Title/Abstract])) OR (T1D[Title/Abstract])) OR (T2D[Title/Abstract])) OR (IDDM[Title/Abstract])) OR (NIDDM[Title/Abstract]) |
| #3 | ("Diabetes Mellitus"[Mesh]) OR (((((((((Diabetes Mellitus[Title/Abstract]) OR (diabetes[Title/Abstract])) OR (diabetic[Title/Abstract])) OR (diabetic[Title/Abstract])) OR (diabet*[Title/Abstract])) OR (T1D[Title/Abstract])) OR (T2D[Title/Abstract])) OR (IDDM[Title/Abstract])) OR (NIDDM[Title/Abstract])) |
| #4 | (((((((systemic immune-inflammation index[Title/Abstract]) OR (systemic immune inflammation index[Title/Abstract])) OR (systemic Immunity-inflammation Index[Title/Abstract])) OR (systemic Immunity inflammation Index[Title/Abstract])) OR (systemic Immunity-inflammation Index[Title/Abstract])) OR (systemic immune inflammatory index[Title/Abstract])) OR (neutrophil×platelets/lymphocyte[Title/Abstract])) OR (SII[Title/Abstract]) |
| #5 | (("Diabetes Mellitus"[Mesh]) OR (((((((((Diabetes Mellitus[Title/Abstract]) OR (diabetes[Title/Abstract])) OR (diabetic[Title/Abstract])) OR (diabetic[Title/Abstract])) OR (diabet*[Title/Abstract])) OR (T1D[Title/Abstract])) OR (T2D[Title/Abstract])) OR (IDDM[Title/Abstract])) OR (NIDDM[Title/Abstract]))) AND ((((((((systemic immune-inflammation index[Title/Abstract]) OR (systemic immune inflammation index[Title/Abstract])) OR (systemic Immunity-inflammation Index[Title/Abstract])) OR (systemic Immunity inflammation Index[Title/Abstract])) OR (systemic Immunity-inflammation Index[Title/Abstract])) OR (systemic immune inflammatory index[Title/Abstract])) OR (neutrophil×platelets/lymphocyte[Title/Abstract])) OR (SII[Title/Abstract])) |

**2.Cochrane**

| Search number | Query |
| --- | --- |
| #1 | MeSH descriptor: [Diabetes Mellitus] explode all trees |
| #2 | (Diabetes Mellitus):ti,ab,kw OR (diabetes):ti,ab,kw OR (diabetic):ti,ab,kw OR (diabets):ti,ab,kw OR (diabet*):ti,ab,kw |
| #3 | (T1D):ti,ab,kw OR (T2D):ti,ab,kw OR (IDDM):ti,ab,kw OR (NIDDM):ti,ab,kw |
| #4 | #1 OR #2 OR #3 |
| #5 | (systemic immune-inflammation index):ti,ab,kw OR (systemic immune inflammation index):ti,ab,kw OR (systemic Immunity-inflammation Index):ti,ab,kw OR (systemic Immunity inflammation Index):ti,ab,kw OR (systemic Immunity-inflammation Index):ti,ab,kw |
| #6 | (systemic immune inflammatory index):ti,ab,kw OR (SII):ti,ab,kw |
| #7 | #5 OR #6 |
| #8 | #4 AND #7 |

**3.Embase**

| Search number | Query |
| --- | --- |
| #1 | 'diabetes mellitus'/exp |
| #2 | 'diabetes mellitus':ab,ti OR diabetes:ab,ti OR diabetic:ab,ti OR diabets:ab,ti OR diabet*:ab,ti OR t1d:ab,ti OR t2d:ab,ti OR iddm:ab,ti OR niddm:ab,ti |
| #3 | #1 AND #2 |
| #4 | #1 OR #2 |
| #5 | 'systemic immune inflammation index'/exp |
| #6 | 'systemic immune-inflammation index':ab,ti OR 'systemic immune inflammation index':ab,ti OR 'systemic immunity inflammation index':ab,ti OR 'systemic immunity-inflammation index':ab,ti OR 'systemic immune inflammatory index':ab,ti OR 'neutrophil×platelets/lymphocyte':ab,ti OR sii:ab,ti |
| #7 | #5 OR #6 |
| #8 | #4 AND #7 |

**4.Web of science**

| Search number | Query |
| --- | --- |
| #1 | Diabetes Mellitus (Topic) OR diabetes (Topic) OR diabetic (Topic) OR diabets (Topic) OR diabet* (Topic) OR T1D (Topic) OR IDDM (Topic) OR NIDDM (Topic) OR T2D (All Fields) |
| #2 | systemic immune-inflammation index (Topic) OR systemic immune inflammation index (Topic) OR systemic Immunity-inflammation Index (Topic) OR systemic Immunity inflammation Index (Topic) OR systemic Immunity-inflammation Index (Topic) OR systemic immune inflammatory index (Topic) OR neutrophil×platelets/lymphocyte (Topic) OR SII (Topic) |
| #3 | #1 AND #2 |

**5. The following table shows an example of a search strategy for Chinese databases (Sinomed).**

| Search number | Query |
| --- | --- |
| #1 | "糖尿病"[不加权:扩展] |
| #2 | "糖尿病"[常用字段:智能] |
| #3 | (#2) OR (#1) |
| #4 | ( "全身免疫炎症指数"[常用字段:智能] OR "系统性免疫炎症指数"[常用字段:智能] OR "血小板计数×中性粒细胞计数/淋巴细胞计数"[常用字段:智能]) |
| #5 | (#4) AND (#3) |
