# Supplementary material for: Association of systemic immune-inflammation index with type 2 diabetes mellitus and its prognostic significance: a systematic review and meta-analysis
Source: Front Endocrinol (Lausanne). 2025 Oct 9;16:1572089. doi: 10.3389/fendo.2025.1572089 (PMC12548759; doi:10.3389/fendo.2025.1572089)
Supplement: Supplementary file 2 [file Table1.docx]

Supplementary Table 1: Characteristics of the included studies.

| **First Author** | **Year** | **Country** | **Study design** | **Sample size** | **Sex**  **Male/female** | **Age (years)** | **Disease background** | **Duration of diabetes** | **Cut-off of SII** | **Follow-up time** | **Study outcomes** | **Confounders** |
| --- | --- | --- | --- | --- | --- | --- | --- | --- | --- | --- | --- | --- |
| Li et al. | 2024 | China | Cross-sectional | 1073 | T2DM:  398/263 DKD:  164/248 | T2DM: 62.58±10.12 DKD:  64.68±11.52 | T2DM | T2DM:  9. 66±7. 43 DKD:  11. 71±8. 68 | Q1:＜329. 89 Q2: 329. 89~450. 55 Q3: 450. 56~634. 51 Q4:＞634. 51 | NM | DKD | ①②③④⑤⑥ |
| Lu et al. | 2024 | China | Prospective | 170 | 101/69 | 60.81±8.21 | AMI+T2DM | 5.00 (3.00,8.00) | Continuous variable | 1 year | MACE | ②⑦ |
| Chang et al. | 2023 | China | Cross-sectional | 668 | 415/253 | 49.42±8.68 | T2DM | <6or≥6 years | 390 | NM | DR | ⑥⑧⑨ |
| Zhang et.al. | 2023 | China | Cross-sectional | 172 | Group1:  45/41 Group2:  46/40 | Group1:  ≤ 60 (n=38)  >60 (n=30） Group2:  ≤ 60 (n= 56)  >60 (n=48) | T2DM | NM | Continuous variable | NM | Cerebral infarction | IMT 、PSV 、EDV 、NLR |
| Dai et.al. | 2024 | China | Retrospective | 158 | 83/75 | 70.79±5.57 | T2DM | Group1:  10 (5,18) Group1:  5 (2,11) | Continuous variable | NM | CAD | ⑧⑨⑩⑪⑫⑬⑭ |
| Urbanowicz et.al. | 2022 | Switzerland | Retrospective | 510 | DM Group: 42/133  NDM Group: 57/278 | DM Group:  67 (61–73) Non-DM Group:  64 (59–70) | T2DM | NM | 958 | 3.7±1.5 years | Mortality | ⑮⑯⑰ |
| Guo et.al. | 2022 | China | Cross-sectional | 3937 | 2040 /1897 | Non-DKD:  56.08 ± 0.39  DKD :  64.20 ± 0.55 | T2DM | NM | 445.21 | NM | DKD | ⑥⑨⑱⑲⑳㉑㉒㉓㉔㉕㉖㉗㉘㉝ |
| Dascalu et.al. | 2023 | Romania | Retrospective | 129 | 67 /62 | 65.6±8.9 | T2DM | 8.9 ± 3.8 | Continuous variable | NM | DR | ⑬⑭ |
| Aljuraiban et.al. | 2024 | Saudi Arabia | Retrospective | 3895 | 1585/2310 | 48.6±18.6 | T2DM | NM | Q1 < 267.4 Q2: 267.4 ~400.7 Q3: 400.7~<588.5 Q4≥588.5 | NM | T2DM | ③⑨ |
| Chen et.al. | 2023 | China | Cross-sectional | 221 | 131/90 | 60 (50, 67) | PDAC | NM | Continuous variable | NM | T2DM | ⑱ |
| Guo et.al. | 2024 | China | Cross-sectional | 9250 | 4827/4423 | 45 (32-61) | Insulin resistance | NM | Q1: 1.53~356.67 Q2: 356.67~552.75 Q3: ≥552.75 | NM | T2DM | ㉜ |
| Li et.al. | 2023 | China | Cross-sectional | 1460 | 716/744 | 59.73±11.31 | T2DM | 7.61±6.37 | Q1: 71.16~328.44  Q2: 328.49~478.92  Q3: 479.89~735.59 Q4: 741.09~7173.95 | NM | Peripheral neuropathy | ⑧⑱⑲㉔㉕㉜㉟㊱ |
| Luo et.al. | 2024 | China | Retrospective | 2111 | 1637 /474 | 65.2±12.2 | Myocardiainfarction | NM | Q1: <613.57  Q2: 613.57~1136.56  Q3: ≥1136.56 | 2.5-5.1 years | T2DM | ⑩⑫⑰⑱⑲㉓㉔㉕㉗㉘ |
| Meng et.al. | 2024 | China | Cross-sectional | 4972 | 2576/2396 | 59.03  (58.61,59.45) | T2DM | NM | 983.5714 | 69  (54-123 )  months | Mortality | ⑱⑲⑳㉑㉒㉓㉔㉕㉚㉛㉜ |
| Tang et.al. | 2024 | China | Prospective | 45,454 | 22,531/22,923 | 47.35± 0.19 | T2DM | NM | Q1: < 337.84 Q2: 377.84 ~ 473.43 Q3: 473.43 ~ 664.70  Q4: > 664.70 | 9.89±0.08 years | Mortality | ①⑨⑱⑲⑳㉑㉒㉓㉔㉕㉙ |
| Wang et.al. | 2023 | China | Retrospective | 500 | NDR:  164/92 DR:  179/65 | NDR:  59 (49, 66) DR:  60 (52.25, 66) | DR | NDR:  7(2, 13.75) DR:  14(9,20) | Q1: <309.6599 Q2:309.6599~415.1037 Q3: 415.1037~539 Q4: >539.7453 | NM | DR | ⑥⑧⑨⑱⑳㉗㉓㉔㉚㉛㉜㉝ |
| Xu et.al. | 2023 | China | Retrospective | 84,645 | 63786/20,859 | 63.0 ±11.0 | CAD | NM | Q1: ≤443.5 Q2: 443.5 ~772.6 Q3: >772.6 | NM | CAD | ⑮⑯⑰⑱⑲㉔㉕ |
| Yan et.al. | 2024 | China | Cross-sectional | 9643 | Quartile 1:  1439 /971 Quartile 2:  1382 /1029 Quartile 3:  1255 /1156 Quartile 4:  1149 /1262 | Quartile 1:  46.4 ±17.1 Quartile 2:  46.2 ±16.7 Quartile 3:  47.1± 16.9 Quartile 4:  49.2 ±17.5 | T2DM | NM | Q1:≤ 322.36 Q2: 322.36 ~447.70 Q3: 447.70 ~633.11 Q4: > 633.11 | 105 months | T2DM | ⑱⑲⑳㉑㉒㉓㉔㉕㉖㉙ |
| Yang et.al. | 2024 | China | Cross-sectional | 8697 | 4475/4222 | 58.95 ±0.19 | T2DM | <3 years (n=1256) 3-10 years (n=3942) > 10 Year (n=3499) | Q1: <345.0 Q2: 345.0~487.5  Q3: 487.5~702.6 Q4: ≥702.6 | 94.8-249 months | Mortality | ⑧⑨⑱⑲⑳㉑㉒㉓㉔㉕㉖㉙㉛ |
| Zhang, et.al. | 2024 | China | Cross-sectional | 6412 | 3332/3080 | 59.36 ± 0.25 | T2DM | NM | Q1: ≤338.46  Q2: 338.47~478.34  Q3: 478.35~692.12  Q4: ≥692.13 | NM | Mortality | ⑱⑲⑳㉑㉓㉖㉙㉞ |
| Bian et.al. | 2023 | China | Prospective | 8,602 | 6611/1991 | 58.41±10.19 | T2DM | NM | 502.5 | 2.4  (2.2–2.6 )  years | CAD | ⑩⑱⑲㉔㉕㉛ |

Notes: NM, not mentioned; Data are expressed as mean ± SD or median (IQR).

Abbreviations: SD, standard deviation; IQR, interquartile range; MACE, major adverse cardiovascular events; SII, systematic immune-inflammation index; T2DM, type 2

diabetes mellitus;CAD,Coronary artery disease;DKD,Diabetic kidney disease;DR,Diabetic retinopathy;NDR,Not diabetic retinopathy;AMI,Acute myocardial infarction;NDM,NOT diabetes mellitus; DM,diabetes mellitus.

①FPG;②TyG;③Fib;④D‑D;⑤FDP;⑥EGFR;⑦BNP;⑧Course of disease;⑨HbA1c;⑩CRP;⑪NEU;⑫CTnT;⑬NLR:NEU/LYM;⑭PLR:PLT/LYM;⑮Stroke;⑯Peripheral artery disease;⑰Left ventricle ejection fraction (LVEF);⑱Age;⑲Sex;⑳Race;㉑Poverty income;㉒Education levels;㉓Smoking status;㉔Hypertension;㉕Hyperlipidemia;㉖BMI;㉗SII;㉘DKD;㉙Drinking;㉚BUN;㉛Scr;㉜Insulin resistance;㉝ALB;㉞Marital status;㉟Body mass;㊱Glomerular filtration.
