# Supplementary material for: Association of systemic immune-inflammation index with type 2 diabetes mellitus and its prognostic significance: a systematic review and meta-analysis
Source: Front Endocrinol (Lausanne). 2025 Oct 9;16:1572089. doi: 10.3389/fendo.2025.1572089 (PMC12548759; doi:10.3389/fendo.2025.1572089)
Supplement: Supplementary file 3 [file Table2.docx]

| **Study** | **Selection** | | | | **Comparability** | **Outcome** | | | **Quality scores** |
| --- | --- | --- | --- | --- | --- | --- | --- | --- | --- |
|  | Is the case definition adequate? | Case representation | Contrast selection | Definition of contrast | Comparability of cohorts on the basis of the design or analysis^&^ | Determination of exposure | Whether case and control exposures were determined using the same method | Nonresponse rate |  |
| Dai 2024 | **＊** | **＊** | **＊** | **＊** | **＊** | **＊** | **＊** | **-** | 7 |
| Urbanowicz 2022 | **＊** | **＊** | **＊** | **＊** | **＊** | **＊** | **＊** | **＊** | 8 |
| Dascalu 2023 | **＊** | **＊** | **＊** | **＊** | **＊** | **-** | **＊** | **＊** | 7 |
| Aljuraiban 2024 | **＊** | **＊** | **＊** | **＊** | **＊** | **＊** | **＊** | **＊** | 8 |
| Luo 2024 | **＊** | **＊** | **＊** | **＊** | **＊＊** | **＊** | **＊** | **＊** | 9 |
| Wang 2023 | **＊** | **＊** | **＊** | **＊** | **＊＊** | **＊** | **＊** | **＊** | 9 |
| Xu 2023 | **＊** | **＊** | **＊** | **＊** | **＊＊** | **＊** | **＊** | **-** | 8 |

Table 2-1:Quality assessment of included studies (Newcastle Ottawa Scale)

Table2-2 ：Quality assessment of included studies (Newcastle Ottawa Scale)

| **Study** | **Selection** | | | | **Comparability** | **Outcome** | | | **Quality scores** |
| --- | --- | --- | --- | --- | --- | --- | --- | --- | --- |
|  | Representativeness of the exposed cohort | Selection of the nonexposed cohort | Ascertainment of exposure | Demonstration that outcome of interest was not present at start of study | Comparability of cohorts on the basis of the design or analysis^&^ | Assessment of outcome | Was follow-up long enough for outcomes to occur^#^ | Adequacy of follow up of cohorts^¶^ |  |
| Lu 2024 | **＊** | **＊** | **＊** | **＊** | **＊** | **＊** | **＊** | **＊** | 8 |
| Bian 2023 | **＊** | **＊** | **＊** | **＊** | **＊＊** | **＊** | **＊** | **＊** | 9 |
| Tang 2024 | **＊** | **＊** | **＊** | **＊** | **＊＊** | **＊** | **-** | **-** | 7 |

**Table 2-3. Joanna Briggs Institute Critical Appraisal tool for cross-sectional study.**

| Study | **Q1** | **Q2** | **Q3** | **Q4** | **Q5** | **Q6** | **Q7** | **Q8** | **Quality** |
| --- | --- | --- | --- | --- | --- | --- | --- | --- | --- |
| Li 2024 | Yes | Yes | Yes | Yes | No | No | Yes | Yes | Moderate |
| Chang 2023 | Yes | Yes | Yes | Yes | No | No | Yes | Yes | Moderate |
| Zhang 2023 | Yes | Yes | Yes | Yes | Yes | Yes | Yes | Yes | High |
| Guo 2022 | Yes | No | Yes | Yes | Yes | Yes | Yes | Yes | High |
| Chen 2023 | Yes | Yes | Yes | Yes | Yes | Yes | Yes | Yes | High |
| Guo 2024 | Yes | No | Yes | Yes | Yes | Yes | Yes | Yes | High |
| Li 2023 | No | Yes | Yes | Yes | Yes | Yes | Yes | Yes | High |
| Meng 2024 | Yes | No | Yes | Yes | Yes | Yes | Yes | Yes | High |
| Yan 2024 | No | No | Yes | Yes | Yes | Yes | Yes | Yes | Moderate |
| Yang 2024 | Yes | No | Yes | Yes | Yes | Yes | Yes | Yes | High |
| Zhang 2024 | Yes | No | Yes | Yes | Yes | Yes | Yes | Yes | High |
| The JBI Checklist provides quality criteria for prevalence studies in eight distinct items. The table indicates which items have been fulfilled for each included study, respectively:  Q1: Were the criteria for inclusion in the sample clearly defined?  Q2: Were the study subjects and the setting described in detail?  Q3: Was the exposure measured in a valid and reliable way?  Q4: Were objective, standard criteria used for measurement of the condition?  Q5: Were confounding factors identified?  Q6: Were strategies to deal with confounding factors stated?  Q7: Were the outcomes measured in a valid and reliable way?  Q8: Was appropriate statistical analysis used? | | | | | | | | | |
