# Supplementary material for: Association of systemic immune-inflammation index with type 2 diabetes mellitus and its prognostic significance: a systematic review and meta-analysis
Source: Front Endocrinol (Lausanne). 2025 Oct 9;16:1572089. doi: 10.3389/fendo.2025.1572089 (PMC12548759; doi:10.3389/fendo.2025.1572089)
Supplement: Supplementary file 4 [file Table3.docx]

**Supplementary Table 3. Subgroup and regression analysis was performed for major adverse cardiovascular events (MACE) and death based on age, sex, study design, sample size, and cut-off of SII**

| Outcomes | Subgroups | Numbers | HR(95%CI) | Heterogeneity | | Regression model |
| --- | --- | --- | --- | --- | --- | --- |
|  |  |  |  | I^2^ | P | P |
| MACE | Age | | | | | |
|  | age<50 | 3 | 0.91 (0.63, 1.31) | 0.0% | 0.621 | ＜0.01 |
|  | age>50 | 18 | 1.65 (1.48, 1.83) | 57.0% | ＜0.01 |  |
|  | Sex(Male%) | | | | | |
|  | Sex(Male%)<70% | 9 | 1.42 (1.19, 1.69) | 9.2% | ＜0.01 | ＜0.01 |
|  | Sex(Male%)>70% | 12 | 1.66 (1.45, 1.90) | 70.5% | ＜0.01 |  |
|  | Study design | | | | | |
|  | Cross-sectional study | 11 | 1.52 (1.32, 1.76) | 17.9% | ＜0.01 | ＜0.01 |
|  | Prospective study | 4 | 1.45 (1.28, 1.65) | 0.0% | ＜0.01 |  |
|  | Retrospective study | 6 | 1.83 (1.47, 2.28) | 80.1% | ＜0.01 |  |
|  | Sample size | | | | | |
|  | Sample size>5000 | 16 | 1.51 (1.32, 1.73) | 68.8% | ＜0.01 | ＜0.01 |
|  | Sample size<5000 | 5 | 1.78 (1.50, 2.11) | 0.0% | ＜0.01 |  |
|  | Cut-off of SII | | | | | |
|  | Cut-off of SII<600 | 4 | 1.45 (1.28, 1.65) | 0.0% | ＜0.01 | ＜0.01 |
|  | Cut-off of SII>600~700 | 5 | 1.29 (0.96, 1.74) | 42.4% | 0.087 |  |
|  | Cut-off of SII>700 | 7 | 1.71 (1.40, 2.09) | 75.7% | ＜0.01 |  |
|  | Cut-off of SII>900 | 5 | 1.78 (1.50, 2.11) | 0.0% | ＜0.01 |  |
| Mortality rate | Age | | | | | |
|  | age<50 | 3 | 0.91 (0.63, 1.31) | 0.0% | 0.621 | ＜0.01 |
|  | age>50 | 14 | 1.74 (1.54, 1.97) | 57.0% | ＜0.01 |  |
|  | Sex(Male%) | | | | | |
|  | Sex(Male%)<60% | 12 | 1.54 (1.33, 1.79) | 20.6%, | ＜0.01 | ＜0.01 |
|  | Sex(Male%)>60% | 5 | 1.80 (1.44, 2.25) | 83.4% | ＜0.01 |  |
|  | Study design | | | | | |
|  | Cross-sectional study | 11 | 1.52 (1.32, 1.76) | 17.9% | ＜0.01 | ＜0.01 |
|  | Retrospective study | 6 | 1.83 (1.47, 2.28) | 80.1% | ＜0.01 |  |
|  | Sample size | | | | | |
|  | Sample size<5000 | 5 | 1.78 (1.50, 2.11) | 0.0% | ＜0.01 | ＜0.01 |
|  | ample size>5000 | 12 | 1.55 (1.31, 1.84) | 72.5% | ＜0.01 |  |
|  | Cut-off of SII | | | | | |
|  | Cut-off of SIII>600~700 | 5 | 1.29 (0.96, 1.74) | 42.4% | 0.087 | ＜0.01 |
|  | Cut-off of SII>700 | 7 | 1.71 (1.40, 2.09) | 75.7% | ＜0.01 |  |
|  | Cut-off of SII>900 | 5 | 1.78 (1.50, 2.11) | 0.0% | ＜0.01 |  |
