# Supplementary figures and images for: Association of systemic immune-inflammation index with type 2 diabetes mellitus and its prognostic significance: a systematic review and meta-analysis
Source: Front Endocrinol (Lausanne). 2025 Oct 9;16:1572089. doi: 10.3389/fendo.2025.1572089 (PMC12548759; doi:10.3389/fendo.2025.1572089)

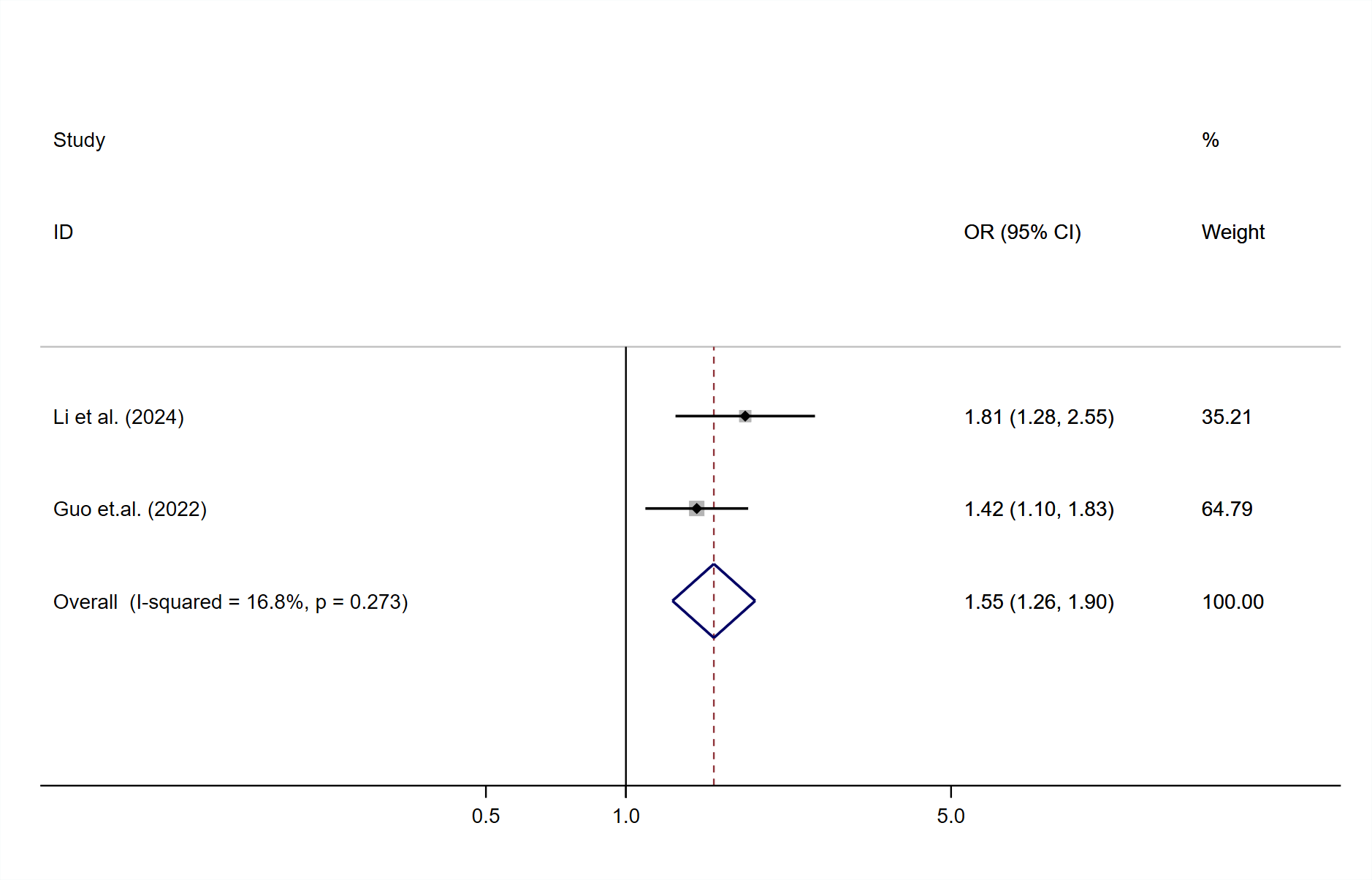

Supplement: Supplementary file 6 [file Image2.tif]

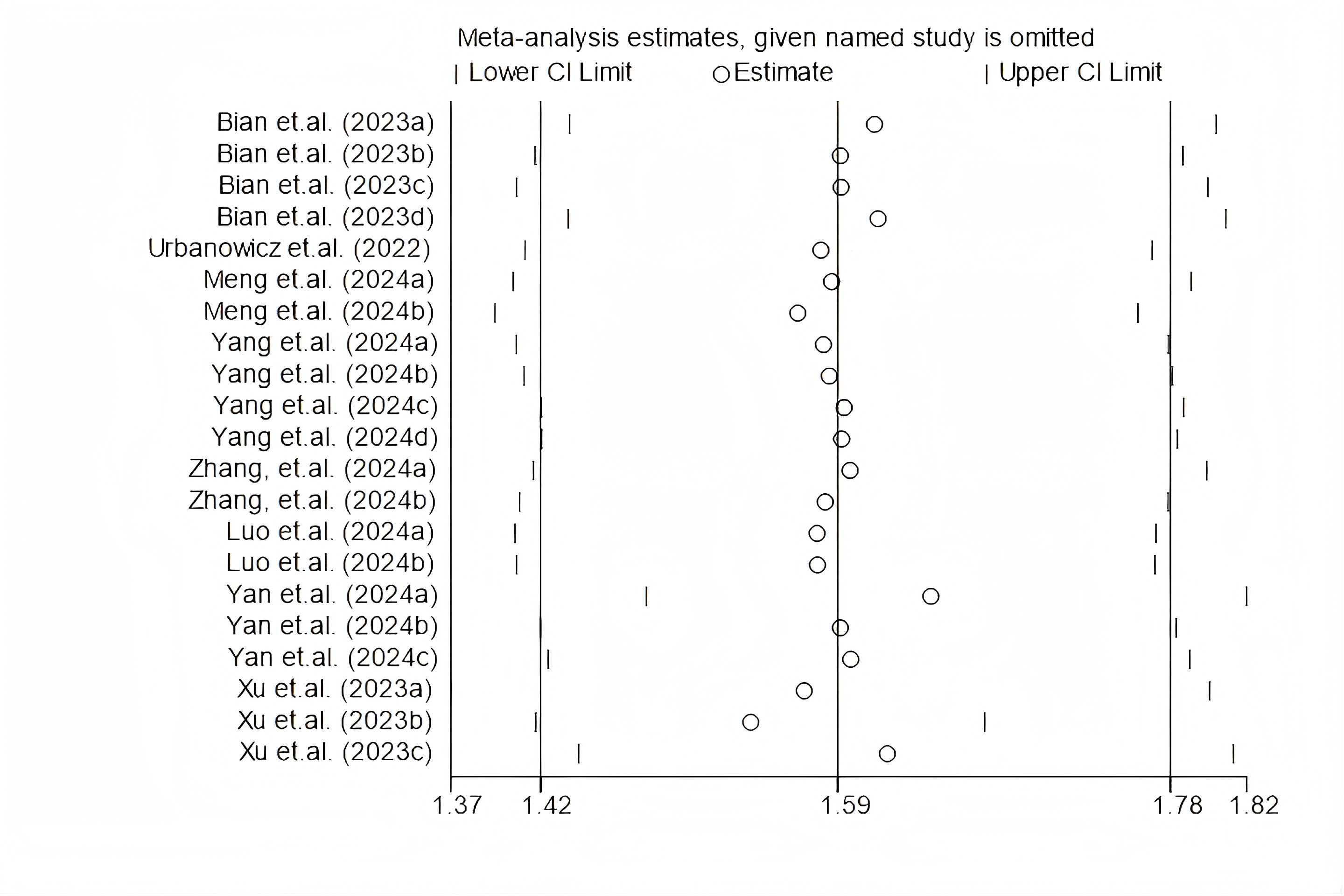

Supplement: Supplementary file 7 [file Image3.jpeg]

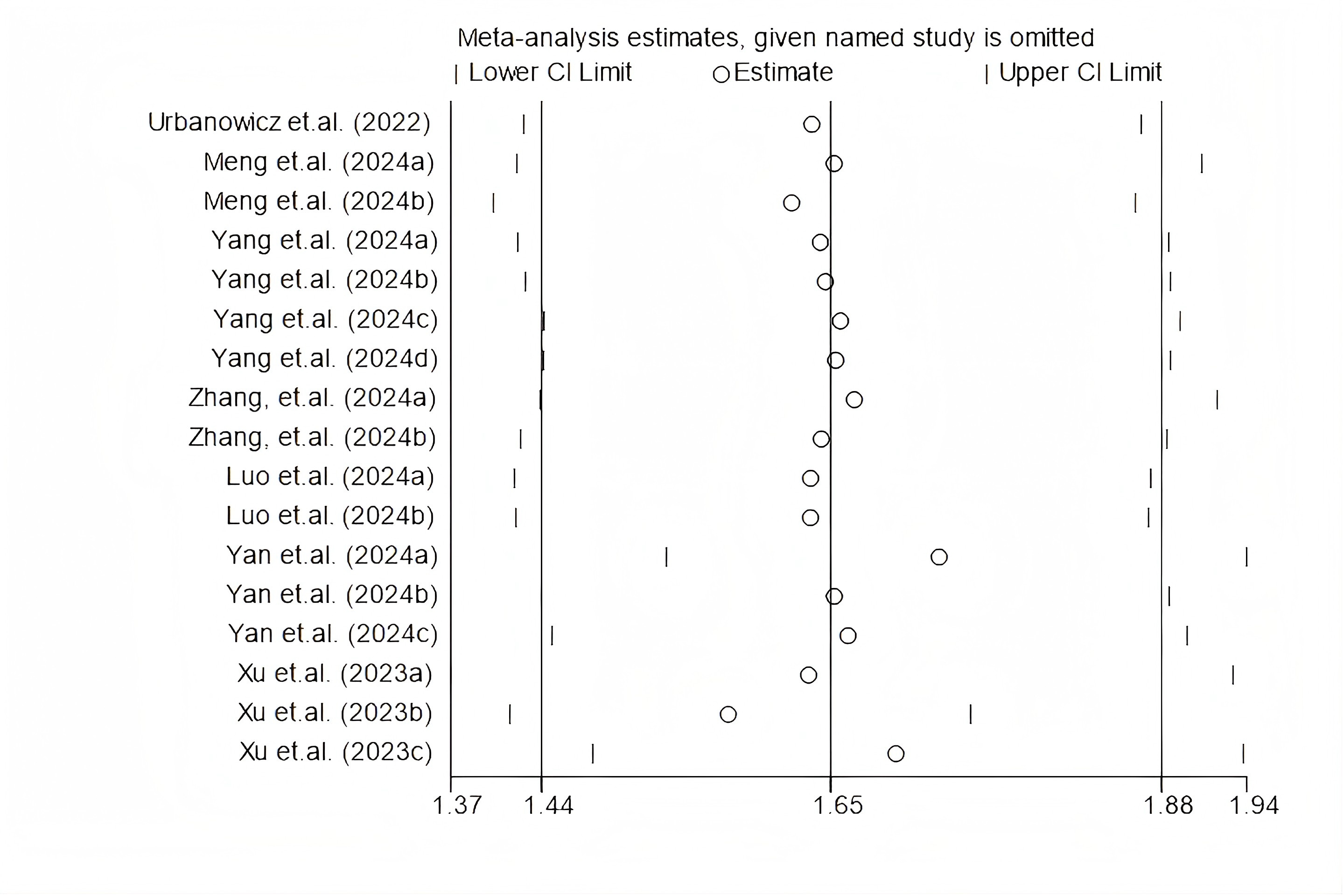

Supplement: Supplementary file 8 [file Image4.jpeg]

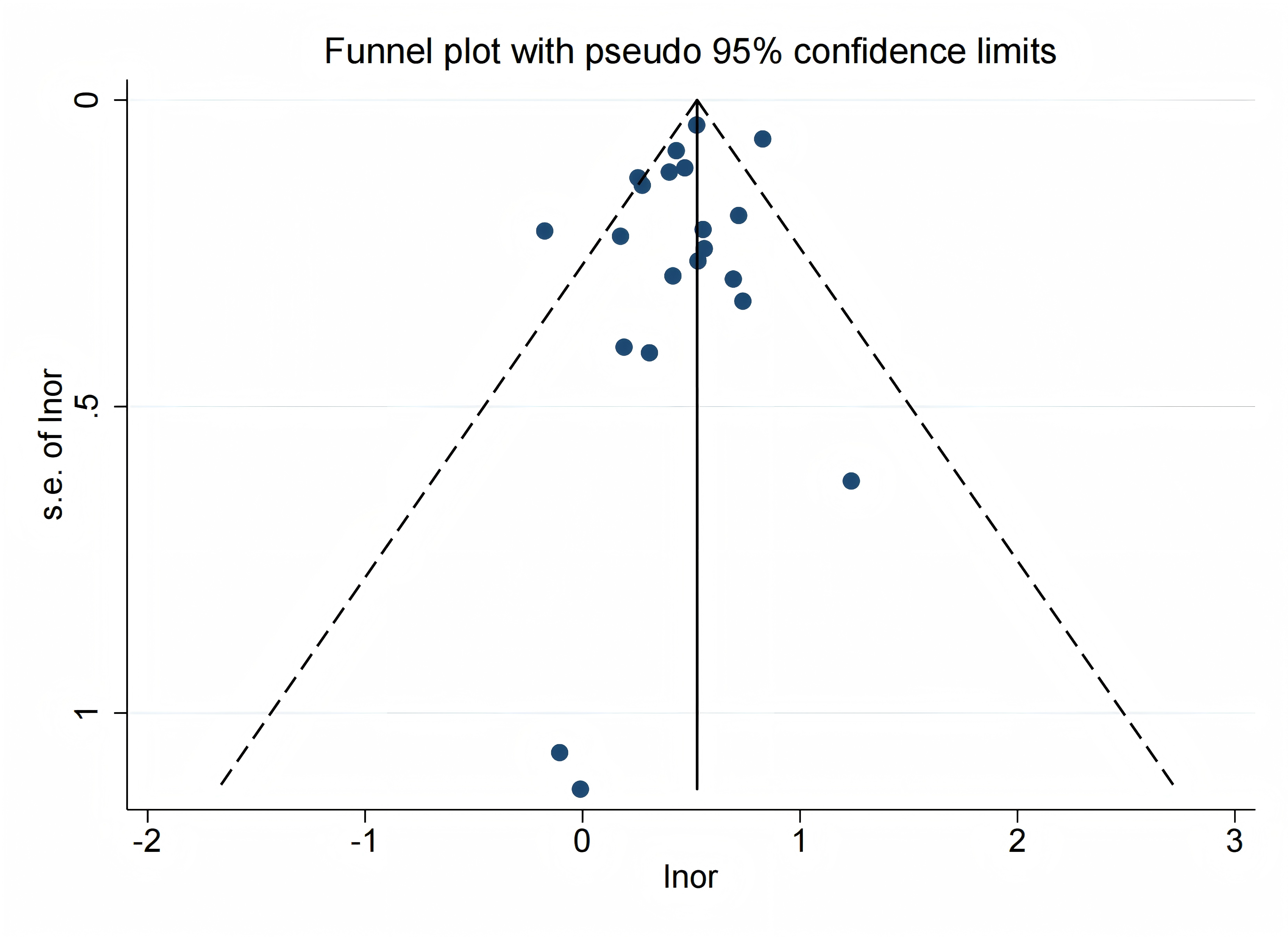

Supplement: Supplementary file 9 [file Image5.jpeg]

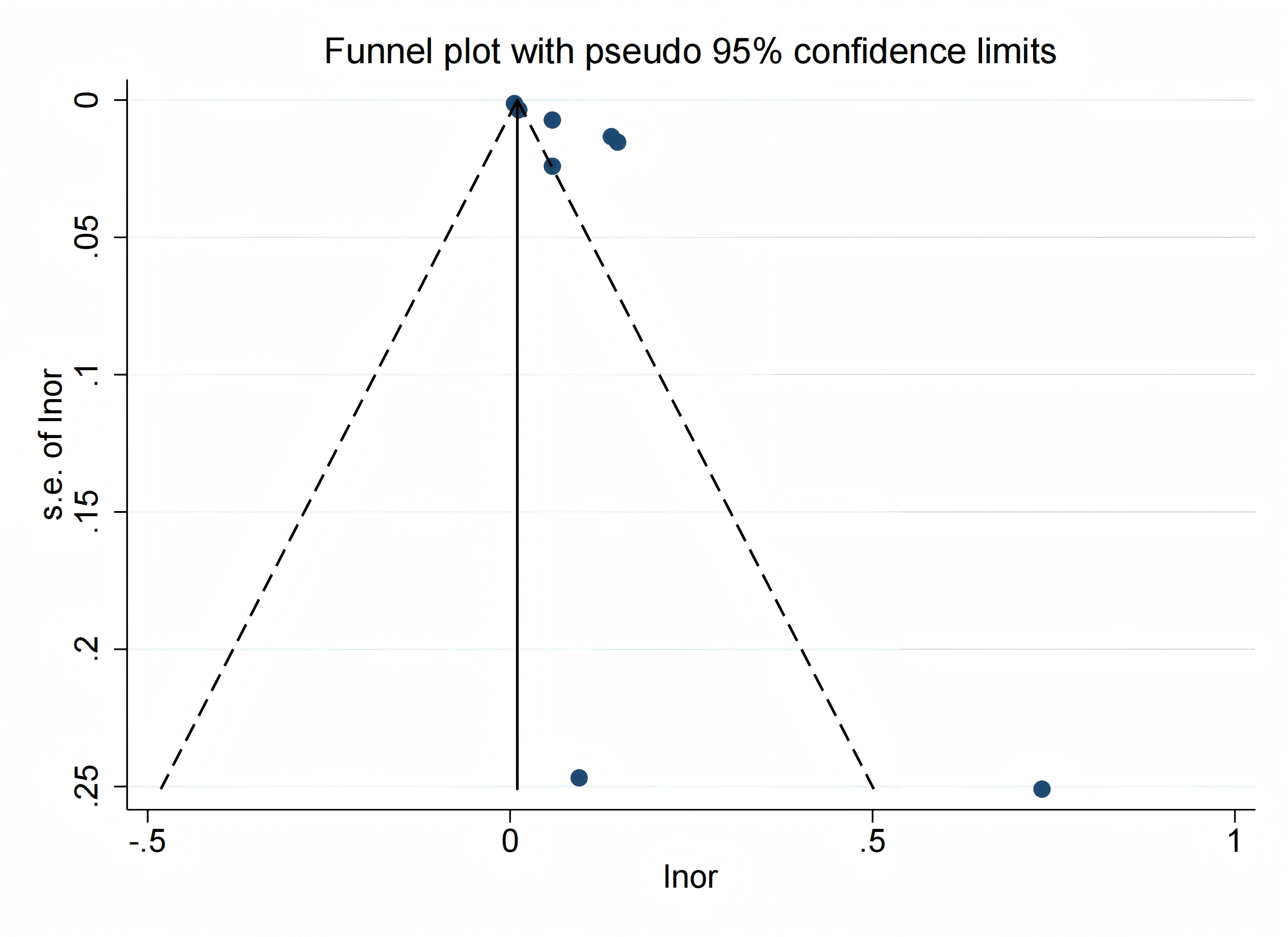

Supplement: Supplementary file 10 [file Image6.jpeg]
